# Dimeric ethyl­tin(IV)–dibromide–hydroxide–*N*,*N*-di­methyl­formamide

**DOI:** 10.1107/S2056989024003268

**Published:** 2024-04-26

**Authors:** Christopher Jan Klecker, Hans Reuter

**Affiliations:** a Chemistry, Osnabrück University, Barbarastrasse 7, 49069 Osnabrück, Germany; University of Massachusetts Dartmouth, USA

**Keywords:** crystal structure, monoorganotin(IV), dihalide, hydroxide, hydrolysis, *trans*-strengthening, hydrogen bonds, DMF

## Abstract

Di-μ-hydroxido-bis­[di­bromido­(di­methyl­formamide-κ*O*)ethyl­tin(IV)], [Sn_2_Br_4_(C_2_H_5_)_2_(OH)_2_(C_3_H_7_NO)_2_], was prepared from ethyl­tin(IV) bromide and *N*,*N*-di­methyl­formamide (DMF) in air. The crystal structure exhibits the typical structural features of dimeric Lewis-base-stabilized monoorganotin(IV)–dihalide–hydroxides, *R*SnHal_2_(OH), *i.e.* two octa­hedrally coordinated Sn atoms are linked together *via* two bridging hydroxide groups, resulting in a centrosymmetric four-membered rhomboid-like Sn–OH ring with acute angles at the Sn atom, obtuse angles at the O atoms and two different tin–oxygen bond lengths. With the shorter bond *trans* to the ethyl group, this observation underlines once more the so-called *trans-strengthening* effect in monoorganotin(IV) com­pounds with octa­hedrally coordinated Sn atoms. Differences and similarities in the bond lengths and angles in the four-membered Sn–OH rings have been worked out for the rings in dimeric diorganotin(IV)–halide–hydroxides, [*R*_2_SnHal(OH)]_2_, and hydrates of dimeric tin(IV)–trihalide–hydroxide–aqua–hydrates, [SnHal_3_(OH)(H_2_O)]_2_·*n*H_2_O.

## Abstract 2

The title com­pound exhibits the typical rhomboid-like four-membered Sn–OH ring found in all dimeric mol­ecules of monoorganotin(IV)–dihalide–hydroxides, with acute bond angles at the Sn atom, obtuse bond angles at O atoms and shorter Sn—O bond lengths *trans* to the ethyl groups.

## 1. Chemical context

The title com­pound ethyl­tin(IV)–dibromide–hydroxide *N*,*N*-di­methyl­formamide solvate, [EtSnBr_2_(OH)·DMF]_2_, belongs to the class of *monoorganotin(IV)–dihalide–hydroxides*, *R*SnHal_2_(OH), representing the first hydrolysis products of the corresponding *monoorganotin(IV)*–*trihalides*, *R*SnHal_3_. Since the basic work of Lecomte *et al.* (1976 ▸), it has been well established that this class of com­pounds crystallizes as dimeric Lewis base (LB)–Brønstedt base (BB)-stabilized adducts, [*R*SnHal_2_(OH)LB]_2_·*n*BB. Depending on LB and BB, four different subclasses of dimeric *monoorganotin(IV)–dihalide–hydroxides* can be distinguished: (**i**) the subclass of dimeric *dihalide–hydroxide–aqua com­plexes*, [*R*SnHal_2_(OH)(H_2_O)]_2_, with LB = H_2_O and *n* = 0, (**ii**) the subclass of *dihalide–hydroxide–solvate com­plexes*, [*R*SnHal_2_(OH)LB]_2_, with LB other than H_2_O and *n* = 0, (**iii**) the subclass of *dihalide–hydroxide–aqua–hydrates*, [*R*SnHal_2_(OH)(H_2_O)]_2_·*n*BB, with LB = BB = H_2_O, and (**iv**) the subclass of *dihalide–hydroxide–aqua–solvates*, [*R*SnHal_2_(OH)(H_2_O)]_2_·*n*BB, with LB = H_2_O and BB = other than H_2_O. 
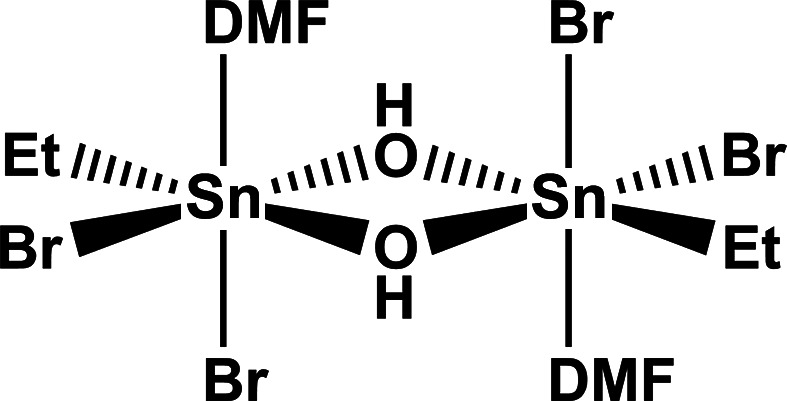


Up to now, the solid-state structures of subclass **i** have been described for Hal = Cl and *R* = Et (Lecomte *et al.*, 1976 ▸), *R* = ^*i*^Pr, ^*i*^Bu (Puff & Reuter, 1989 ▸), *R* = ^*n*^Bu (Holmes *et al.*, 1988 ▸) and *R* = (6,6-di­methyl­bicyclo[3.1.1]hept-2-yl)methyl (Beckmann *et al.*, 2009 ▸), but for subclass **ii**, only the crystal structure of the isobutyl com­pound with Hal = Cl and LB = DMF (Reuter & Ye, 2013 ▸) is known. In the case of subclass **iii**, only the single-crystal structure determination of the methyl com­pound with Hal = Cl and *n* = 3 (Johnson & Knobler, 1994 ▸) exists and for subclass **iv**, the *n*-butyl com­pounds with Hal = Cl, BB = methyl­benzo­thia­zole and *n* = 4 (Wei, 1994 ▸), and BB = dimethyl cyano­carbonodi­thio­imidate and *n* = 4 (Mbaye *et al.*, 2023 ▸) or *n* = 2 (Diop *et al.*, 2022 ▸) are available.

The title com­pound, belonging to subclass **ii**, was found accidentally as a hydrolysis product of humid air during an attempt to synthesize a com­plex of ethyl­tin(IV)–tribromide with DMF and represents the first structurally characterized *monoorganotin(IV)–hydroxide–dihalide* with bromine as the halide.

## 2. Structural commentary

**Figure 1:**
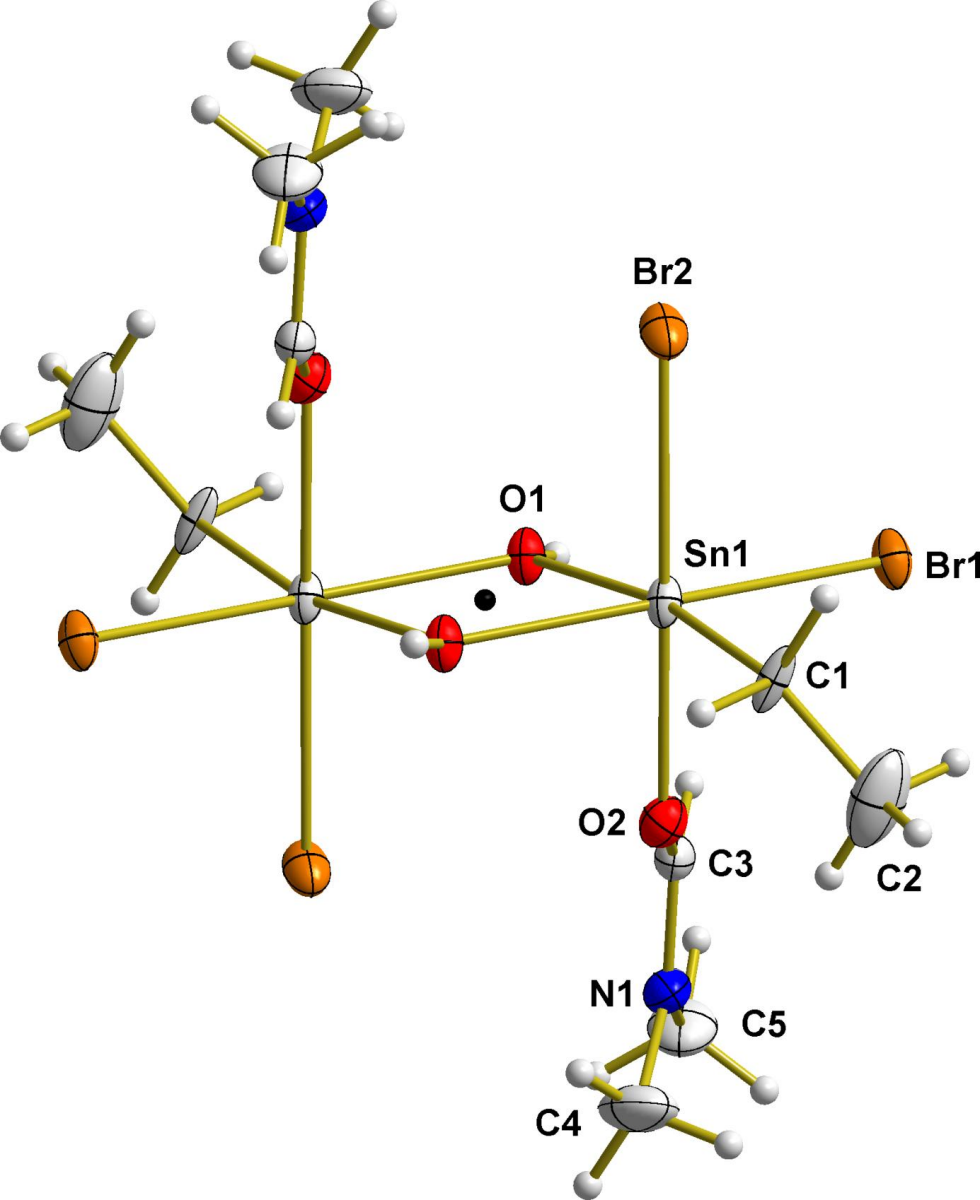
Displacement ellipsoid plot of the dimeric centrosymmetric mol­ecule found in the crystal of [EtSnBr_2_(OH)·DMF]_2_, showing the atom numbering of the asymmetric unit. With the exception of the H atoms, which are shown as spheres of arbitrary radius, all other atoms are drawn with displacement ellipsoids at the 40% probability level.

The title com­pound crystallizes in the monoclinic space group *P*2_1_/*c*, as was unambiguously confirmed from systematic absence conditions. The unit cell contains two dimeric centrosymmetric mol­ecules (Fig. 1 ▸), resulting in half a mol­ecule in the asymmetric unit. The mol­ecule exhibits the typical structural features of the *monoorganotin(IV)–dihalide–hy­dro­xides*, *i.e.* two octa­hedrally coordinated Sn atoms are linked together *via* two bridging hydroxide groups whereby a planar four-membered Sn–OH ring results.

**Figure 2:**
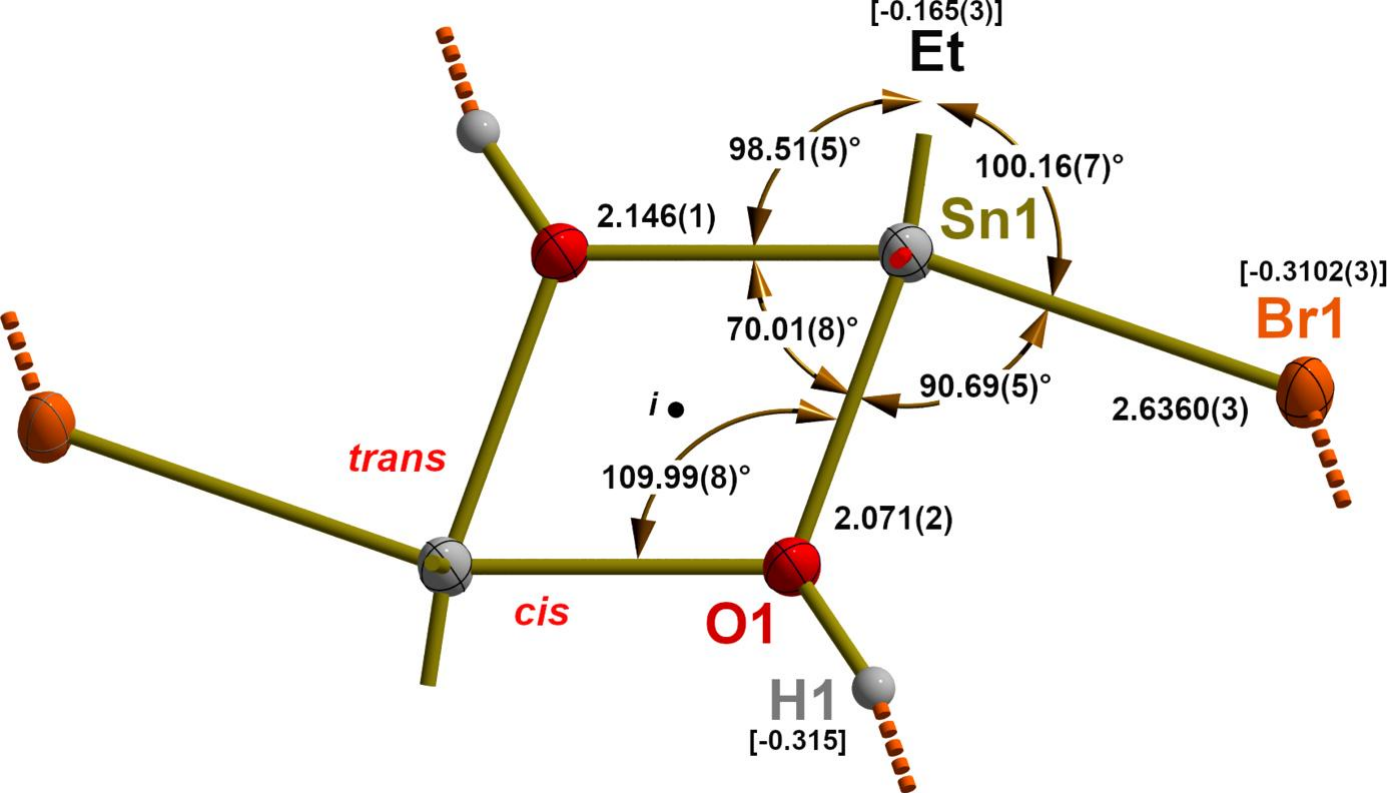
Displacement ellipsoid plot of the centrosymmetric four-membered tin–oxygen ring of the [EtSnBr_2_(OH)·DMF]_2_ mol­ecule, highlighting selected bond lengths (Å), angles (°) and distances (Å) from the Sn–O reference plane in square brackets. With the exception of the H atoms, which are shown as spheres of arbitrary radius, all other atoms are drawn with displacement ellipsoids at the 40% probability level. For clarity, ethyl groups are stripped down to the Sn—C bonds drawn as shortened sticks. Inter­molecular O—H⋯Br hydrogen bonds are indicated as dashed sticks in brown. Descriptors *trans* and *cis* refer to the position of the corres­ponding bonds with respect to the tin–carbon bond of the ethyl group.

This Sn–OH ring (Fig. 2 ▸) has a characteristic rhomboid-like shape with acute [70.01 (8)°] angles at the Sn atoms, obtuse angles [109.99 (8)°] at the O atoms and two distinct different tin–oxygen bond lengths [2.071 (2) and 2.1461 (1) Å], the shorter of which is opposite to the organic group. This kind of bond-length shortening, designated in the literature as *trans*-strengthening (Paseshnitchenko *et al.*, 1985 ▸; Buslaev *et al.*, 1989 ▸), is typically found in the case of monoorganotin(IV) com­pounds with tin in a sixfold octa­hedral coordination.

Four-membered Sn–OH rings are structure-dominating features in many organic and inorganic tin(IV) com­pounds. Thus, they occur, for example, in the dimeric *diorganotin(IV)–halide–hydroxides*, [*R*_2_SnHal(OH)]_2_, with trigonal-bipyrami­dally coordinated Sn atoms. There the bond angles are in the same order; different Sn—O bond lengths, however, result from the *axial* and *equatorial* positions of the hydroxide groups within the trigonal-bipyramidal coordination of the Sn atoms (*cf*. Reuter, 2022 ▸). A somewhat different geometry is observed in the case of the four-membered Sn–OH rings of the dimeric *tin(IV)–trihalide–hydroxide–aqua com­plexes*, [SnHal_3_(OH)(H_2_O)]_2_, where the Sn atoms are also octa­hedrally coordinated. These com­pounds constitute the pure inorganic equivalents of the class of com­pounds discussed here with an additional halide atom instead of the organic group *R*. In analogy to the dimeric *monoorganotin(IV)–dihalide–aqua–com­plexes*, these inorganic counterparts can be divided into similar subclasses. For Hal = Br, the structures of only two polymorphs (Howie *et al.*, 2005 ▸; de Lima *et al.*, 2010 ▸) of a hydrate (subclass **iii**), with 3.5 additional water mol­ecules, are actually known. In both, the dimeric mol­ecules are noncentrosymmetric and the Sn–OH rings are not planar, but only slightly buckled. Nevertheless, these rings exhibit a geometry with similar bond angles at the oxygen [mean value: 108.4 (5)°, 4 data points] and the Sn atoms [mean value: 71.6 (3)°, 4 data points], but the Sn—O bond lengths become more equal [2.081 (1)–2.072 (8) Å] so that the rings adopt a more rhombus-like shape.

The C—C distance [C1—C2 = 1.485 (5) Å] in the ethyl group is to some extent shorter than the value of 1.513 (14) Å evaluated by Allen *et al.* (1987 ▸) for the mean distance between two *sp* ^3^-hybridized C atoms. This deviation is probably caused by atom vibration, as indicated by the displacement ellipsoids (Fig. 1 ▸). The Sn—C distance [Sn—C = 2.228 (2) Å] is enlarged com­pared to the sum (2.15 Å) of the normal co­va­lent radii (Cordero *et al.*, 2008 ▸) of tin (1.39 Å) and carbon (0.76 Å), but is in the same order of magnitude as the Sn—C bond length [2.20 (3) Å] found in [EtSnCl_2_(OH)·H_2_O]_2_ (Lecomte *et al.*, 1976 ▸). Much shorter tin–carbon bonds [2.139 (4) and 2.130 (4) Å] have been reported for the corresponding DMF com­pound with *R* = ^*i*^Bu and Hal = Cl (Reuter & Ye, 2013 ▸).

Both tin–bromine bonds are of different lengths with the longer one [2.6360 (3) Å] in the case of the *in-plane* (ip) Br1 atom and the shorter one [2.5893 (4) Å] in the case of the *out-of-plane* (oop) Br2 atom. The reason for this obviously arises from the fact that the first is involved in a hydrogen bond with the hydroxide group of a neighbouring mol­ecule (see below), while the second is only involved in van der Waals inter­actions. It is notable that both values are markedly longer (0.069 and 0.080 Å) than the tin–bromine distances in the above-mentioned *tin(IV)–tribromide–hydroxide–aqua–hy­drates* [mean Sn—Br_ip_ = 2.509 (5) Å, 8 data points; mean Sn—Br_oop_ = 2.567 (14) Å, 4 data points].

**Figure 3:**
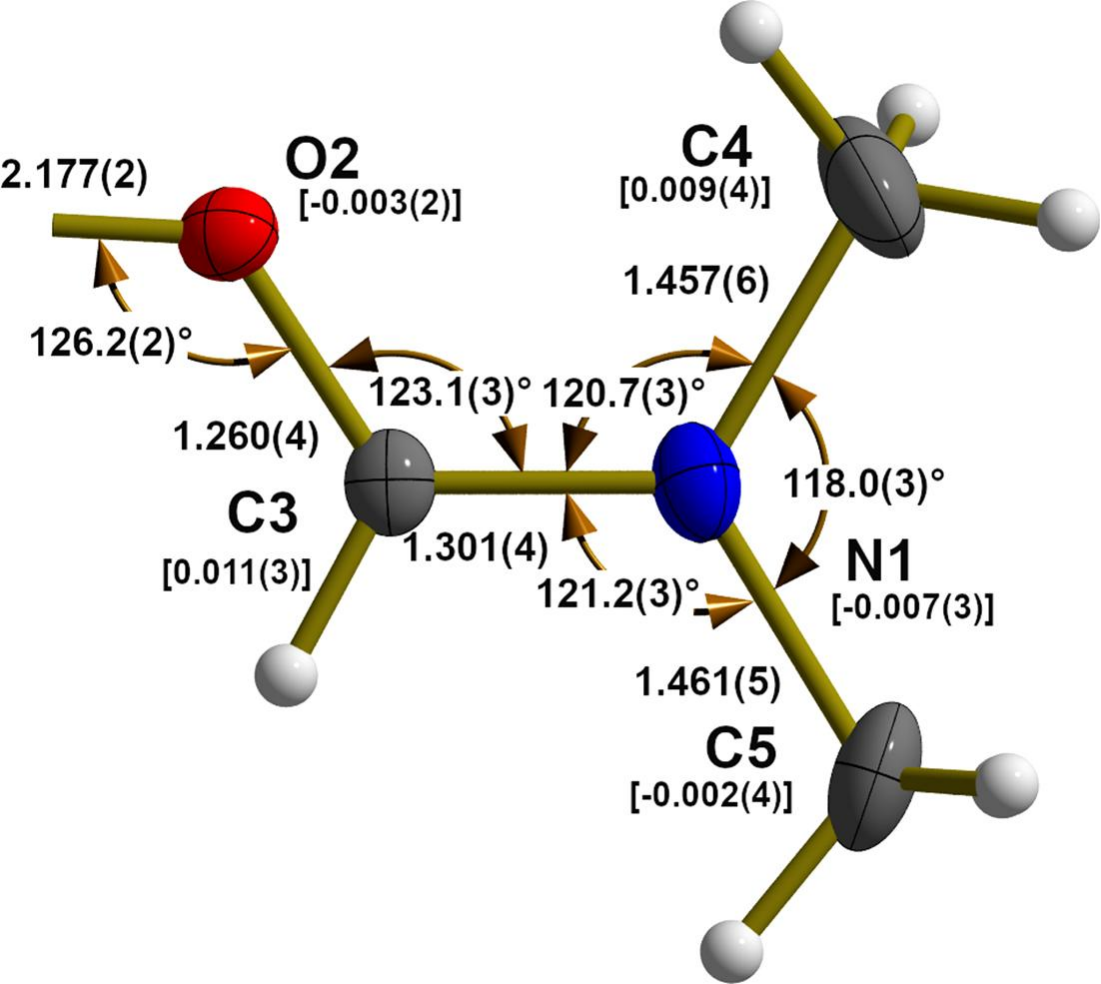
Displacement ellipsoid plot of the DMF mol­ecule, with selected bond lengths (Å), angles (°) and distances (Å) from the least-squares plane through the non-H atoms in square brackets. The dative Sn⋯O bond is indicated as a shortened stick.

The coordinated DMF mol­ecule is almost planar, as the distances of the O, C and N atoms from the least-squares plane indicate (Fig. 3 ▸). The coordinative bond has a length of 2.177 (2) Å, while the bond angle at the O atom is 126.2 (2)°. Both values differ significantly from the corresponding values [2.210 (3)/2.202 (4) Å and 120.8 (3)/124.8 (4)°] observed in the noncentrosymmetric mol­ecules of [^*i*^BuSnCl_2_(OH)(DMF)]_2_ (Reuter & Ye, 2013 ▸). The angle between the least-squares plane through the non-H atom of the DMF mol­ecule and the Sn—O_DMF_ bond length is 3.12 (8)°.

Structural distortion of the DMF mol­ecule as a result of its coordinative bond to the Sn atom is well expressed and concerns not only the bond lengths but also the bond angles. Structural data for pure DMF have been determined twice (Borrmann *et al.*, 2000 ▸; Ratajczyk *et al.*, 2019 ▸) under normal pressure and at a temperature of 100 K. Both crystallize in the triclinic space group *P* 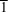 , with two different mol­ecules in the asymmetric unit. As the individual structure parameters within both mol­ecules and between the different measurements differ to some extent, in the following, the mean values of each four data points are used. Most notable are the changes in bond lengths: thus, the carbon–oxygen distance increases by 0.031 Å from 1.229 (2) Å in pure DMF to 1.260 (4) Å in the coordinated molecule; simultaneously, the carbon–nitro­gen distance decreases by 0.038 Å from 1.339 (2) to 1.301 (4) Å, while the distances between the methyl C atoms and the N atoms remain mostly unaffected [*cis*-CH_3_—N(pure/coordinated) = 1.453 (2)/1.457 (6) Å and *trans*-CH_3_—N(pure/coordinated) = 1.454 (2)/1.461 (5) Å]. The greatest changes of the bond angles are observed for O—C—N, decreasing by 2.3° from 125.4 (2)° in pure DMF to 123.1 (3)° in the coordinated mol­ecule, and to a smaller extent (0.8°) for CH_3_—N—CH_3_, increasing from 117.2 (3) to 118.0 (3)°. The changes of the CH—N—CH_3_ angles range from −0.4 to −0.5°.

## 3. Supra­molecular features

**Figure 4:**
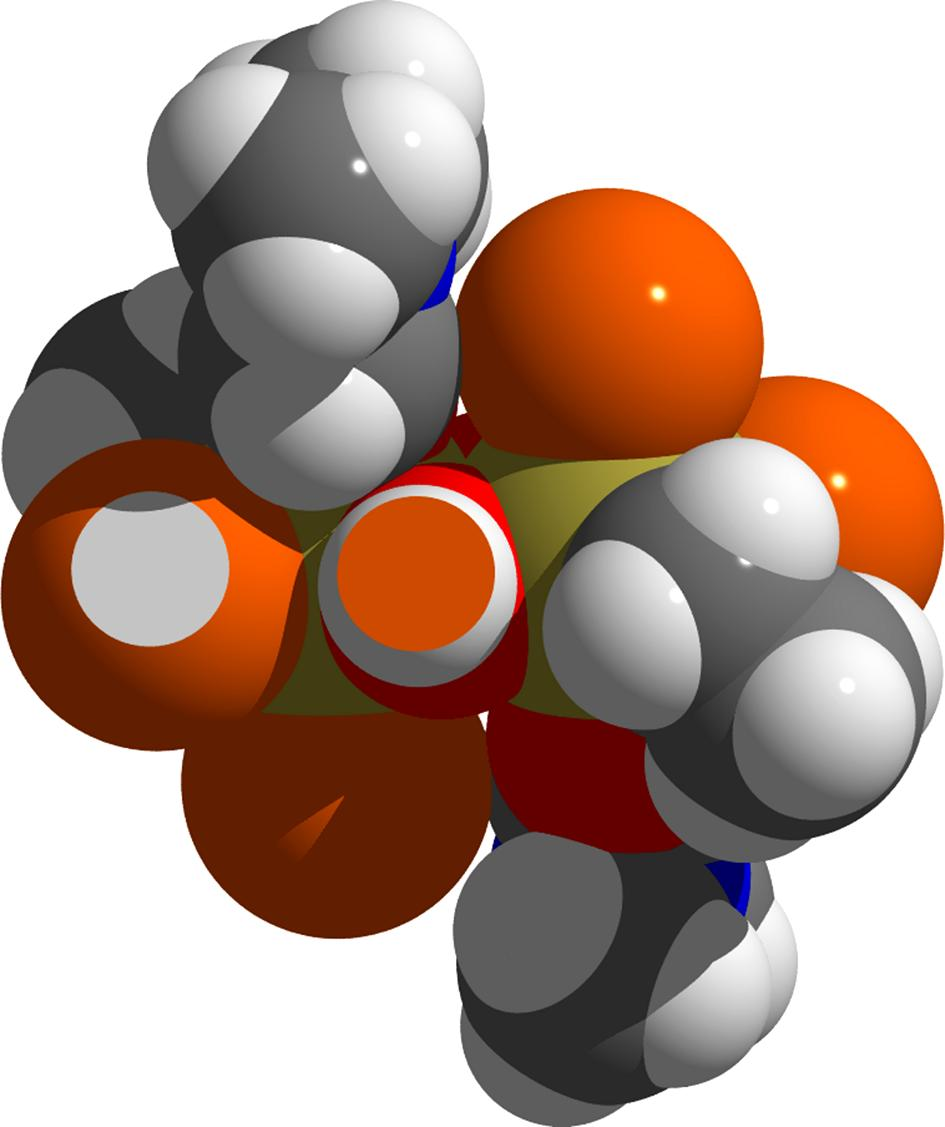
Space-filling model of the [EtSn(OH)Br_2_·DMF]_2_ mol­ecule, showing the overlap of the H and Br atoms in the region of the hydrogen-bridging bond. These atoms are visualized as truncated two-coloured spheres. Atom colours and van der Waals radii (Å) are as follows: Br = brown/1.83, H = white/1.10, C = grey/1.70, O = red/1.52, N = blue/1.55 and Sn = brass/2.17.

**Figure 5:**
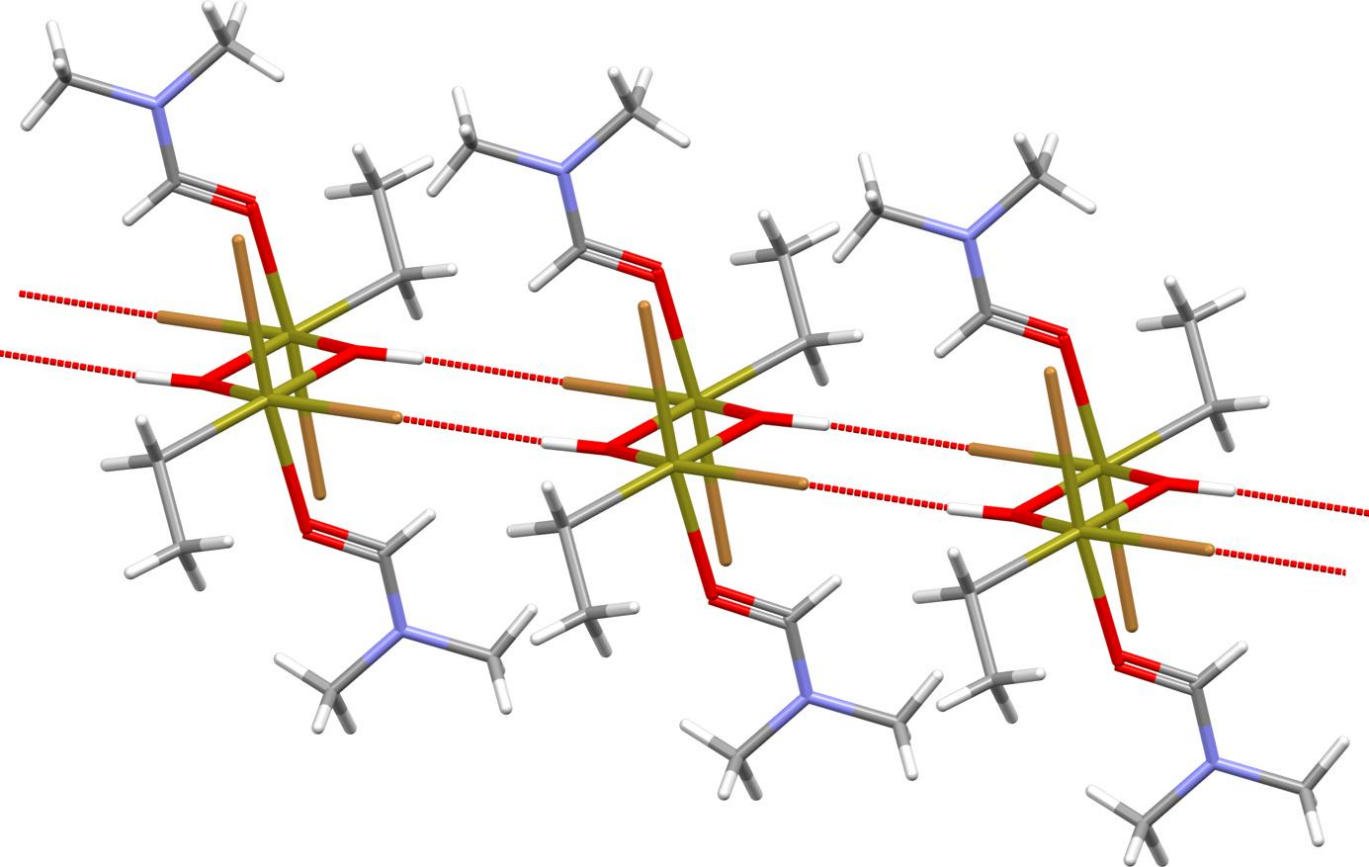
Stick-model showing in detail the chain-like arrangement of the [EtSn(OH)Br_2_·DMF]_2_ mol­ecules resulting from inter­molecular O—H⋯Br hydrogen bonds (red dashed sticks). The image shows three com­plete mol­ecules with their hydrogen bonds to neighbouring mol­ecules. Two-coloured sticks based on atom colours are as follows: Br = brown, H = white, C = grey, O = red, N = blue and Sn = brass

**Figure 6:**
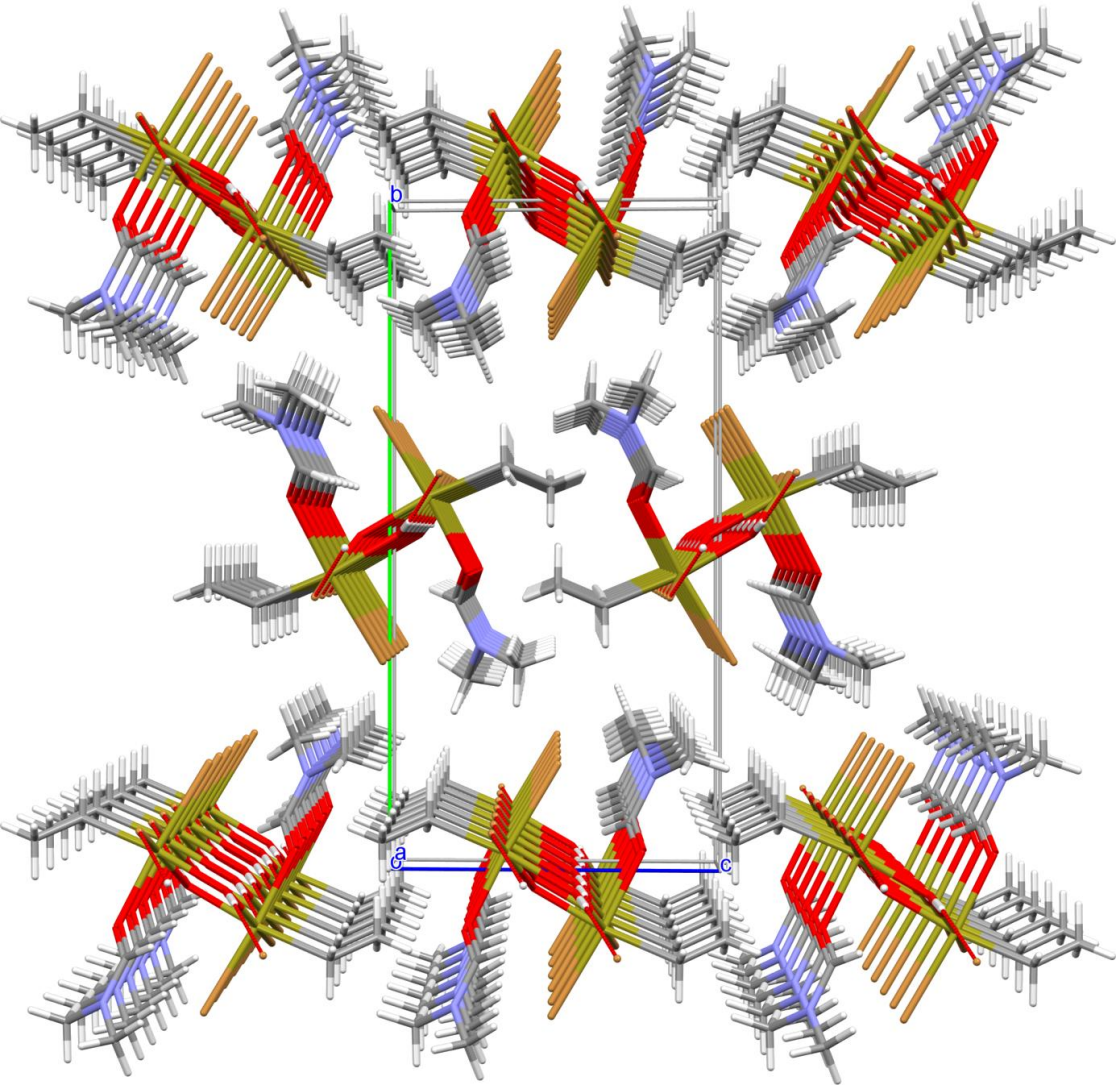
Perspective view into the crystal structure of [EtSn(OH)Br_2_·DMF]_2_ looking down the crystallographic *a* axis and showing the arrangement of the chains of hydrogen-bonded [EtSn(OH)Br_2_·DMF]_2_ mol­ecules in relation to the unit cell (*a* axis = red, *b* axis = green and *c* axis = blue).

In the solid, hydrogen bonds exist between the hydroxide groups and the Br1 atoms of adjacent mol­ecules, as the space-filling model (Fig. 4 ▸) using the van der Waals radii of Mantina *et al.* (2009 ▸) indicates. The resulting chain-like arrangement of the hydrogen-bonded mol­ecules (Fig. 5 ▸) takes place in the direction of the crystallographic *a* axis. With a donor–acceptor distance of 3.283 (2) Å between the Br and O atoms, they rank as strong. The bridging angle at the H atom is 164.8°. As the second Br atom (Br2) does not take part in any hydrogen bonds, the inter­actions between the individual chains are confined to van der Waals contacts (Fig. 6 ▸).

## 4. Synthesis and crystallization

In a fumehood, 0.39 g (1 mmol) of ethyl­tin(IV) tribromide, C_2_H_5_Br_3_Sn, prepared from ethyl­tin(IV) trichloride *via* halide exchange with an excess of potassium bromide in dry acetone was mixed with 2 ml *N*,*N*-di­methyl­formamide (DMF) on a petri dish with a glass lid. Crystal formation was checked every day using an optical microscope. The first crystals of the title com­pound appeared after two weeks.

## 5. Refinement

Crystal data, data collection and structure refinement details are summarized in Table 1 ▸. The positions of all H atoms were clearly identified in difference Fourier syntheses. Those of the organic groups were refined with calculated positions (–CH_3_ = 0.96 Å, –CH_2_– = 0.97 Å and –CH– = 0.93 Å) and common *U*_iso_(H) parameters for each individual group. The position of the H atom of the OH group was refined with a fixed O—H distance of 0.96 Å before it was fixed and allowed to ride on the parent O atom with an isotropic displacement parameter.

## Supplementary Material

Crystal structure: contains datablock(s) I, global. DOI: 10.1107/S2056989024003268/yy2010sup1.cif

Structure factors: contains datablock(s) I. DOI: 10.1107/S2056989024003268/yy2010Isup2.hkl

CCDC reference: 2348369

Additional supporting information:  crystallographic information; 3D view; checkCIF report

## Figures and Tables

**Table 1:** Experimental details

Crystal data	
Chemical formula	[Sn_2_Br_4_(C_2_H_5_)_2_(OH)_2_(C_3_H_7_NO)_2_]
*M*_r_	795.35
Crystal system, space group	Monoclinic, *P*2_1_/*c*
Temperature (K)	100
*a*, *b*, *c* (Å)	7.0415 (3), 17.9349 (8), 9.0148 (5)
β (°)	100.812 (2)
*V* (Å^3^)	1118.26 (9)
*Z*	2
Radiation type	Mo *K*α
μ (mm^−1^)	9.39
Crystal size (mm)	0.26 × 0.16 × 0.12

Data collection	
Diffractometer	Bruker APEXII CCD
Absorption correction	Multi-scan (*SADABS*; Krause *et al.*, 2015 ▸)
*T*_min_, *T*_max_	0.514, 0.723
No. of measured, independent and observed [*I* > 2σ(*I*)] reflections	84799, 2687, 2455
*R*_int_	0.037
(sin θ/λ)_max_ (Å^−1^)	0.661

Refinement	
*R*[*F* ^2^ > 2σ(*F* ^2^)], *wR*(*F* ^2^), *S*	0.018, 0.046, 1.07
No. of reflections	2687
No. of parameters	107
H-atom treatment	Only H-atom displacement parameters refined
Δρ_max_, Δρ_min_ (e Å^−3^)	1.07, −0.50

Computer programs: *APEX2* and *SAINT* (Bruker, 2009 ▸), *SHELXS97* (Sheldrick, 2008 ▸), *SHELXL2014* (Sheldrick, 2015 ▸), *DIAMOND* (Brandenburg, 2006 ▸), *Mercury* (Macrae *et al.*, 2020 ▸) and *publCIF* (Westrip, 2010 ▸).

## supplementary crystallographic information

### Crystal data

**Table:**

[Sn_2_Br_4_(C_2_H_5_)_2_(OH)_2_(C_3_H_7_NO)_2_]	*F*(000) = 744
*M**_r_* = 795.35	*D*_x_ = 2.362 Mg m^−^^3^
Monoclinic, *P*2_1_/*c*	Mo *K*α radiation, λ = 0.71073 Å
*a* = 7.0415 (3) Å	Cell parameters from 9635 reflections
*b* = 17.9349 (8) Å	θ = 2.9–29.1°
*c* = 9.0148 (5) Å	µ = 9.39 mm^−^^1^
β = 100.812 (2)°	*T* = 100 K
*V* = 1118.26 (9) Å^3^	Plate, colourless
*Z* = 2	0.26 × 0.16 × 0.12 mm

### Data collection

**Table:**

Bruker APEXII CCD diffractometer	2455 reflections with *I* > 2σ(*I*)
φ and ω scans	*R*_int_ = 0.037
Absorption correction: multi-scan (SADABS; Krause *et al.*, 2015)	θ_max_ = 28.0°, θ_min_ = 3.0°
*T*_min_ = 0.514, *T*_max_ = 0.723	*h* = −9→9
84799 measured reflections	*k* = −23→23
2687 independent reflections	*l* = −11→11

### Refinement

**Table:**

Refinement on *F*^2^	Hydrogen site location: mixed
Least-squares matrix: full	Only H-atom displacement parameters refined
*R*[*F*^2^ > 2σ(*F*^2^)] = 0.018	*w* = 1/[σ^2^(*F*_o_^2^) + (0.0201*P*)^2^ + 2.2958*P*] where *P* = (*F*_o_^2^ + 2*F*_c_^2^)/3
*wR*(*F*^2^) = 0.046	(Δ/σ)_max_ = 0.002
*S* = 1.07	Δρ_max_ = 1.07 e Å^−^^3^
2687 reflections	Δρ_min_ = −0.50 e Å^−^^3^
107 parameters	Extinction correction: SHELXL2014 (Sheldrick, 2015), Fc^*^=kFc[1+0.001xFc^2^λ^3^/sin(2θ)]^-1/4^
0 restraints	Extinction coefficient: 0.00097 (16)
Primary atom site location: structure-invariant direct methods	

### Special details

**Table:**

Geometry. All esds (except the esd in the dihedral angle between two l.s. planes) are estimated using the full covariance matrix. The cell esds are taken into account individually in the estimation of esds in distances, angles and torsion angles; correlations between esds in cell parameters are only used when they are defined by crystal symmetry. An approximate (isotropic) treatment of cell esds is used for estimating esds involving l.s. planes.

### Fractional atomic coordinates and isotropic or equivalent isotropic displacement parameters (Å^2^)

**Table:**

	*x*	*y*	*z*	*U*_iso_*/*U*_eq_	
Sn1	0.13007 (2)	0.55518 (2)	0.14030 (2)	0.01599 (6)	
C1	0.0660 (3)	0.59933 (18)	0.3566 (3)	0.0203 (6)	
H11	0.0714	0.6534	0.3552	0.078 (7)*	
H12	−0.0642	0.5850	0.3656	0.078 (7)*	
C2	0.2042 (6)	0.5712 (3)	0.4897 (4)	0.0549 (12)	
H21	0.2043	0.5177	0.4886	0.078 (7)*	
H22	0.1660	0.5885	0.5805	0.078 (7)*	
H23	0.3316	0.5892	0.4861	0.078 (7)*	
Br1	0.50184 (4)	0.58619 (2)	0.17646 (3)	0.02061 (8)	
Br2	0.04385 (4)	0.67775 (2)	−0.00891 (4)	0.02626 (8)	
O1	0.1412 (2)	0.49890 (10)	−0.0585 (2)	0.0179 (4)	
H1	0.2590	0.4824	−0.0870	0.051 (12)*	
O2	0.2199 (3)	0.44926 (11)	0.2493 (2)	0.0226 (4)	
N1	0.3916 (4)	0.34269 (14)	0.2843 (3)	0.0251 (5)	
C3	0.3503 (4)	0.40693 (15)	0.2192 (3)	0.0204 (5)	
H3	0.4204	0.4223	0.1469	0.056 (5)*	
C4	0.2812 (6)	0.3145 (2)	0.3939 (5)	0.0461 (10)	
H41	0.3403	0.3310	0.4933	0.056 (5)*	
H42	0.2795	0.2610	0.3910	0.056 (5)*	
H43	0.1512	0.3331	0.3697	0.056 (5)*	
C5	0.5454 (5)	0.29569 (19)	0.2467 (4)	0.0390 (8)	
H51	0.6023	0.3198	0.1705	0.056 (5)*	
H52	0.4924	0.2485	0.2095	0.056 (5)*	
H53	0.6425	0.2880	0.3354	0.056 (5)*	

### Atomic displacement parameters (Å^2^)

**Table:**

	*U*^11^	*U*^22^	*U*^33^	*U*^12^	*U*^13^	*U*^23^
Sn1	0.01125 (9)	0.01715 (10)	0.01884 (10)	0.00173 (6)	0.00092 (7)	−0.00585 (7)
C1	0.0071 (10)	0.0454 (17)	0.0086 (12)	0.0054 (11)	0.0023 (9)	−0.0085 (11)
C2	0.039 (2)	0.099 (4)	0.0262 (19)	0.000 (2)	0.0046 (16)	−0.019 (2)
Br1	0.01178 (12)	0.02018 (14)	0.02940 (16)	0.00052 (9)	0.00265 (10)	−0.00779 (11)
Br2	0.02073 (14)	0.01634 (14)	0.03855 (18)	0.00282 (10)	−0.00257 (12)	−0.00237 (11)
O1	0.0115 (8)	0.0210 (10)	0.0212 (10)	0.0010 (7)	0.0031 (7)	−0.0074 (8)
O2	0.0190 (9)	0.0265 (10)	0.0218 (10)	0.0020 (8)	0.0024 (8)	0.0001 (8)
N1	0.0238 (12)	0.0240 (13)	0.0248 (13)	−0.0017 (10)	−0.0028 (10)	0.0061 (10)
C3	0.0200 (13)	0.0194 (13)	0.0195 (14)	−0.0023 (10)	−0.0020 (11)	−0.0005 (11)
C4	0.047 (2)	0.046 (2)	0.046 (2)	−0.0021 (17)	0.0109 (18)	0.0257 (18)
C5	0.0422 (19)	0.0231 (16)	0.048 (2)	0.0112 (14)	−0.0003 (16)	0.0047 (15)

### Geometric parameters (Å, º)

**Table:**

Sn1—O1	2.071 (2)	O1—H1	0.9600
Sn1—O1^i^	2.146 (2)	O2—C3	1.260 (3)
Sn1—O2	2.177 (2)	N1—C3	1.301 (4)
Sn1—C1	2.228 (2)	N1—C4	1.457 (4)
Sn1—Br2	2.5893 (4)	N1—C5	1.461 (4)
Sn1—Br1	2.6360 (3)	C3—H3	0.9300
C1—C2	1.485 (5)	C4—H41	0.9600
C1—H11	0.9700	C4—H42	0.9600
C1—H12	0.9700	C4—H43	0.9600
C2—H21	0.9600	C5—H51	0.9600
C2—H22	0.9600	C5—H52	0.9600
C2—H23	0.9600	C5—H53	0.9600
O1—Sn1^i^	2.146 (2)		
			
O1—Sn1—O1^i^	70.01 (8)	H21—C2—H23	109.5
O1—Sn1—O2	84.87 (8)	H22—C2—H23	109.5
O1^i^—Sn1—O2	85.71 (7)	Sn1—O1—Sn1^i^	109.99 (8)
O1—Sn1—C1	167.78 (9)	Sn1—O1—H1	123.8
O1^i^—Sn1—C1	98.51 (8)	Sn1^i^—O1—H1	121.8
O2—Sn1—C1	90.26 (10)	C3—O2—Sn1	126.16 (18)
O1—Sn1—Br2	90.42 (5)	C3—N1—C4	120.7 (3)
O1^i^—Sn1—Br2	95.65 (5)	C3—N1—C5	121.2 (3)
O2—Sn1—Br2	174.34 (5)	C4—N1—C5	118.0 (3)
C1—Sn1—Br2	94.97 (8)	O2—C3—N1	123.1 (3)
O1—Sn1—Br1	90.69 (5)	O2—C3—H3	118.4
O1^i^—Sn1—Br1	159.59 (5)	N1—C3—H3	118.4
O2—Sn1—Br1	86.02 (5)	N1—C4—H41	109.5
C1—Sn1—Br1	100.16 (7)	N1—C4—H42	109.5
Br2—Sn1—Br1	90.900 (11)	H41—C4—H42	109.5
C2—C1—Sn1	112.1 (2)	N1—C4—H43	109.5
C2—C1—H11	109.2	H41—C4—H43	109.5
Sn1—C1—H11	109.2	H42—C4—H43	109.5
C2—C1—H12	109.2	N1—C5—H51	109.5
Sn1—C1—H12	109.2	N1—C5—H52	109.5
H11—C1—H12	107.9	H51—C5—H52	109.5
C1—C2—H21	109.5	N1—C5—H53	109.5
C1—C2—H22	109.5	H51—C5—H53	109.5
H21—C2—H22	109.5	H52—C5—H53	109.5
C1—C2—H23	109.5		

Symmetry code: (i) −*x*, −*y*+1, −*z*.

### Hydrogen-bond geometry (Å, º)

**Table:**

*D*—H···*A*	*D*—H	H···*A*	*D*···*A*	*D*—H···*A*
O1—H1···Br1^ii^	0.96	2.35	3.283 (2)	165

Symmetry code: (ii) −*x*+1, −*y*+1, −*z*.
